# Vaginal microbiota and functional prediction along the progression of cervical lesions in postmenopausal women

**DOI:** 10.3389/fcimb.2026.1911664

**Published:** 2026-10-02

**Authors:** Yanjia Liu, Cailing Ma, Lijuan Quan, Yan Li, Yanling Zhou, Renqi Zhang, Lipeng Zhang, Yinawati Tuerxun

**Affiliations:** 1 Department of Gynecology, The First Affiliated Hospital of Xinjiang Medical University, Urumqi, China; 2 State Key Laboratory of Pathogenesis, Prevention and Treatment of High Incidence Diseases in Central Asia, Urumqi, China; 3 Xinjiang Key Laboratory of Medical Animal Model Research, Urumqi, China; 4 The Health Management Center of The First Affiliated Hospital of Xinjiang Medical University, Urumqi, China

**Keywords:** cervical cancer, cervical squamous intraepithelial lesion, HPV infection, Nanopore sequencing, postmenopausal women, vaginal microbiota

## Abstract

**Objective:**

To investigate the changes in vaginal microbiota in postmenopausal women from Xinjiang, China, with high-risk human papillomavirus (HPV) infection, cervical squamous intraepithelial lesion, and cervical cancer (CC).

**Methods:**

This study enrolled healthy postmenopausal women (HC group, n=25), postmenopausal women with high-risk HPV infection (HPV group, n=25), low-grade squamous intraepithelial lesion (LSIL group, n=25), high-grade squamous intraepithelial lesion (HSIL group, n=25), and cervical cancer (CC group, n=25). DNA was extracted from vaginal secretions, and full-length 16S rRNA gene sequencing was performed on the Nanopore platform. The EasyAmplicon 2 pipeline was used for sequence processing and taxonomic annotation, PICRUSt2 was used for functional prediction, and R software was used for vaginal microbiota diversity and statistical analyses.

**Results:**

Among five groups of postmenopausal women, Shannon and Simpson alpha diversity indices were comparable, whereas Chao1/ACE richness differed significantly (*p <* 0.05). PERMANOVA showed no ASV-level beta diversity separation, with significant phylum-level divergence (R^2^ = 0.105, *p* = 0.001). *Lactobacillus iners* dominated all samples but decreased in CC, alongside elevated opportunistic pathogens. LEfSe screened group-specific taxa: *L. johnsonii* (HC), *Dialister pneumosintes* (LSIL), *Candidatus Saccharibacteria* (HSIL), and *Streptococcus* spp./*Bacteroides fragilis* (CC). HC vs CC contained the most DABs (35), and LSIL vs HSIL the fewest (13). Co-occurrence network complexity peaked in LSIL/HSIL (41 correlated pairs) and declined in CC (28 pairs). Ninety-two differential KEGG pathways were identified, with 60 enriched in LSIL. *L. iners* displayed dual correlations with diverse metabolic pathways, indicating comprehensive remodeling of vaginal microbiota structure, interactions and functions across progressive cervical lesions.

**Conclusion:**

Vaginal microbiota undergoes progressive dysbiosis during the progression from high-risk HPV infection to CC in postmenopausal women. Distinct microbial alterations are observed at each disease stage, with substantial functional remodeling occurring at the LSIL stage. These findings provide a reference for early intervention and research on microbial biomarkers.

## 1 Introduction

Declining estrogen concentrations in postmenopausal women remodel the vaginal microenvironment, characterized by diminished vaginal epithelial glycogen production and vaginal dryness (NAMS Editorial Panel, 2020). Concomitantly, profound compositional shifts occur within the vaginal microbiota. Previous research has verified that healthy postmenopausal individuals exhibit lower *Lactobacillus* abundance and diversity relative to premenopausal women (Burton and Reid, 2002; Gustafsson et al., 2011; So et al., 2024; Li and Wu, 2025). These microecological disturbances may exacerbate persistent high-risk HPV infection and accelerate the progression of precancerous cervical lesions to invasive cervical carcinoma (Kombe Kombe et al., 2020; Li and Wu, 2025). Accordingly, profiling vaginal microbial signatures across postmenopausal patients stratified by HPV status and hierarchical cervical lesion severity could deliver robust evidence to guide individualized clinical intervention. The majority of current vaginal microbiome investigations depend on short-read sequencing targeting partial variable segments of the 16S rRNA gene, an approach that fails to afford precise, species-level taxonomic resolution (Chen et al., 2018; Zhang et al., 2020; Diao et al., 2021). Critically, congeneric bacterial species often exert divergent physiological effects: certain strains maintain mucosal homeostasis, whereas others correlate with cervical pathological phenotypes, highlighting striking disparities in pathogenic capacity between closely related taxa (Zhang et al., 2020; Diao et al., 2021). Collectively, high-resolution species-level microbial profiling is strongly warranted for genital tract microecology research (Diao et al., 2021; Zeng et al., 2024). Long-read sequencing commercialized by Oxford Nanopore Technologies (ONT) addresses key bottlenecks inherent to conventional short-read workflows (Mahmoud et al., 2024; Zhang et al., 2024; Liu and Conesa, 2025). This platform generates full-length 16S rRNA gene amplicons of approximately 1.5 kb. Owing to its superior taxonomic discriminatory power, Nanopore long reads support accurate species-level annotation, robust phylogenetic tree construction, and comprehensive functional inference of polymicrobial communities (Mahmoud et al., 2024). Herein, we applied ONT full-length 16S rRNA gene sequencing to characterize vaginal microbiome profiles among a cohort of Xinjiang postmenopausal women encompassing five subgroups: healthy HPV/TCT-negative controls, patients with isolated high-risk HPV infection, low-grade squamous intraepithelial lesions (LSIL), high-grade squamous intraepithelial lesions (HSIL), and invasive cervical cancer (CC). This study aimed to delineate stepwise shifts in vaginal community composition, intertaxon co-occurrence networks, and microbial functional capacity along the full spectrum of cervical lesion progression in this local postmenopausal population.

## 2 Materials and methods

### 2.1 Study participants

Postmenopausal women treated in the gynecological department of the First Affiliated Hospital of Xinjiang Medical University from September 2025 to December 2025 were recruited. Inclusion criteria were as follows: (1) The patient has reached menopause (amenorrhea for no less than 1 year, with both female endocrine hormones FSH and LH exceeding 40 IU/L). (2) Sexual history of one year or more. (3) Patients with positive high-risk HPV results underwent colposcopic cervical biopsy or cervical conization at our hospital, and postoperative pathological diagnoses were CC, HSIL, LSIL and chronic cervicitis. (4) Have resided in Xinjiang for more than one year. Exclusion criteria were as follows: (1) Sexual activity within one week prior to collection. (2) A history of vaginal douching or medication use within two weeks preceding collection. (3) Use of antibiotics or sex hormones within one month prior to collection. (4) Past history of hysterectomy, radiotherapy or chemotherapy for genital tract tumors. (5) Other malignant tumor conditions or systemic immune disorders.

### 2.2 Ethical statement

This study was approved by the Ethics Committee of the First Affiliated Hospital of Xinjiang Medical University (approval number: K202504-45). The studies were conducted in accordance with the local legislation and institutional requirements. The participants provided their written informed consent to participate in this study.

### 2.3 Grouping standards

All enrolled postmenopausal participants were divided into five groups according to high-risk HPV status and pathological results. 1) HC group (healthy control group): Healthy physical examination participants with negative high-risk HPV and TCT results and no cervical pathological lesions. 2) HPV group: Patients with positive high-risk HPV and pathologically diagnosed chronic cervicitis, without cervical intraepithelial neoplasia (CIN) or CC, confirmed by colposcopic biopsy or conization. 3) LSIL group: Patients with positive high-risk HPV and pathologically diagnosed LSIL, corresponding to CIN1, confirmed by colposcopic biopsy or conization. 4) HSIL group: Patients with positive high-risk HPV and pathologically diagnosed HSIL, including CIN2 and CIN3, confirmed by colposcopic biopsy or conization. 5) CC group: Patients with positive high-risk HPV who were pathologically confirmed with CC via colposcopic biopsy or cervical conization.

### 2.4 Full-length 16S rRNA gene sequencing and microbiome functional prediction

#### 2.4.1 Sample collection, DNA extraction, and full-length 16S rRNA gene amplification

Vaginal specimens were collected from all participants prior to colposcopic biopsy or cervical conization. Vaginal secretions from the vaginal fornix and lateral vaginal walls were sampled using sterile swabs manufactured by Jiangsu Kangjian. All specimens were transported on ice and preserved at −80 °C within 30 min of collection. Total genomic DNA was isolated with the HiPure Soil DNA Mini Kit (Magen, Cat. No. D3142-03) in strict accordance with the manufacturer’s instructions. DNA purity was determined via Nanodrop ONE, and DNA concentration was quantified using a Qubit 3.0 fluorometer (Thermo Scientific). The integrity of extracted DNA was verified by electrophoresis on a 1% agarose gel (DYY-6C, Beijing Liuyi Biotechnology). Full-length 16S rRNA gene amplification was carried out in a 30 μL reaction system using the VAHTS HiFi Amplification Mix (Vazyme, Cat. No. N618-02). The thermal cycling program was set as follows: initial denaturation at 95 °C for 3 min; 25 cycles consisting of 95 °C for 30 s, 60 °C for 60 s, and 72 °C for 1.5 min; followed by a final extension step at 72 °C for 3 min. Amplicons were purified with Agencourt AMPure XP magnetic beads (Beckman Coulter), subjected to quality verification, and pooled at an equimolar concentration ratio.

#### 2.4.2 Library construction, Oxford Nanopore long-read sequencing, and functional prediction

Sequencing libraries were generated with the SQK-LSK114 kit (Oxford Nanopore Technologies, ONT). Briefly, end repair and dA-tailing were conducted using the NEBNext End Repair/dA-Tailing Module (NEB), followed by sequencing adapter ligation. Purified library fragments were loaded onto R10.4.1 flow cells and sequenced on the PromethION platform (ONT) to acquire full-length 16S rRNA gene long reads. Raw sequencing data underwent quality filtering, ASV clustering, and taxonomic annotation through the EasyAmplicon 2 pipeline. This pipeline exhibits full compatibility with long-read third-generation sequencing amplicon datasets, integrates multiple mainstream analytical tools, and supports integrated one-stop visualization (Liu et al., 2023; Yousuf et al., 2024; Luo et al., 2026). Functional profiling of vaginal microbiota based on ASV sequences was implemented using PICRUSt2 (https://github.com/picrust/picrust2). All subsequent analyses, including α- and β-diversity assessment, statistical hypothesis testing, linear discriminant analysis effect size (LEfSe) for differential taxa screening, microbial co-occurrence network construction, and statistical evaluation of PICRUSt2 functional outputs, were performed in R software (v4.3.1) with RStudio IDE (v4.6.0). Kruskal–Wallis test was adopted for multi-group comparisons, while Wilcoxon rank-sum test was used for pairwise comparisons of microbial abundance.

### 2.5 Statistical analysis

All statistical analyses were conducted using R software (version 4.3.1). The Shapiro-Wilk normality test revealed that continuous variables including age, body mass index (BMI), age at menopause, age at menopause, gravidity and parity were not normally distributed. These variables were presented as median (interquartile range). The Kruskal-Wallis H test was used for overall comparisons across the five groups. Where significant differences were found, *post-hoc* pairwise comparisons were performed using the Dunn test with Bonferroni correction. Categorical variables including ethnicity, residence, marital status and educational level were summarized as counts and percentages, and compared using the chi-square test. At the species level, four pairwise comparisons (HC vs CC, HC vs HSIL, HPV vs CC, LSIL vs HSIL) were performed using the Mann-Whitney U test to assess differences in microbial abundance. The Benjamini-Hochberg false discovery rate (FDR) method was applied for multiple testing correction, and a corrected *q* value < 0.05 was considered statistically significant. Spearman’s correlation analysis was used to evaluate correlations between microbial species, with FDR correction applied likewise. For all other statistical tests, a *p* value < 0.05 was regarded as the threshold for statistical significance.

## 3 Results

### 3.1 Demographic characteristics

A total of 125 postmenopausal women were enrolled, with 25 cases in each of the five groups (HC, HPV, LSIL, HSIL, CC). No significant differences were found in age, BMI, age at menopause, ethnicity, residence and marital status across the groups (all *p* > 0.05, **Table 1**). Significant inter-group differences were observed in years since menopause (H = 10.85, *p* = 0.0283). *Post-hoc* Bonferroni test revealed that the HPV group had a longer menopausal duration than the HSIL group (adjusted *p* = 0.0198), and no other pairwise comparisons reached statistical significance. Gravidity also differed significantly among groups (H = 12.51, *p* = 0.0139). After correction, the HC group presented lower gravidity than the HSIL group (adjusted *p* = 0.0334) and CC group (adjusted *p* = 0.0372), while no significant differences were detected between the remaining groups.

**Table 1:** Basic characteristics of participants.

Parameter	CC(n=25)	HSIL(n=25)	LSIL(n=25)	HPV(n=25)	HC(n=25)	*p* value
Age (years), median(Q1, Q3)	58 (54, 61)	54 (52, 56)	58 (54, 61)	58 (53, 62)	54 (53, 61)	0.2712^a^
BMI (kg/m²), median(Q1, Q3)	24.09 (21.23, 28.91)	24.61 (23.05, 25.78)	26.99 (26.81, 28.04)	26.81 (23.01, 29.78)	24.46 (22.41, 27.34)	0.4945^a^
Age at menopause (years), median(Q1, Q3)	50 (48, 50)	50 (48, 54)	51 (48, 52)	49 (45, 51)	51 (50, 52)	0.2435^a^
Years since menopause (years), median(Q1, Q3)	7 (5, 10)	3 (1, 7)	7 (2, 10)	10 (4, 15)	4 (2, 10)	0.0283^a^
Gravidity, median(Q1, Q3)	4 (3, 5)	4 (3, 6)	3 (2, 4)	3 (3, 3)	3 (2, 3)	0.0139^a^
Parity, median(Q1, Q3)	2 (1, 3)	2 (1, 4)	1 (1, 2)	2 (1, 2)	1 (1, 2)	0.2441^a^
Ethnicity, n(%)						0.606^b^
Han	13 (52.0%)	16 (64.0%)	18 (72.0%)	16(64.0%)	16(64.0%)	
others	12 (48.0%)	9 (36.0%)	7 (28.0%)	9 (36.0%)	9 (36.0%)	
Education level, n(%)						0.410^b^
Bachelor degree or above	1 (4.0%)	0 (0)	0 (0)	0 (0)	0 (0)	
High school or junior college	10 (40.0%)	9 (36.0%)	14 (56.0%)	9 (36.0%)	14 (56.0%)	
Middle school or below	14 (56.0%)	16 (64.0%)	11 (44.0%)	16 (64.0%)	11 (44.0%)	
Residence, n(%)						0.173^b^
Urban	20 (80.0%)	18 (72.0%)	17 (68.0%)	13 (52.0%)	20 (80.0%)	
Rural	5 (20.0%)	7 (28.0%)	8 (32.0%)	12 (48.0%)	5 (20.0%)	
Marital status, n(%)						0.324^b^
Married status, n(%)	20 (80.0%)	21 (84.0)	21 (84.0%)	24 (96.0%)	23 (92.0%)	
Divorced	5 (20.0%)	2 (8.0%)	3 (12.0%)	0 (0)	1 (4.0%)	
Widowed	0 (0)	2 (8.0%)	1 (4.0%)	1 (4.0%)	1 (4.0%)	

Data are presented as median (interquartile range) for continuous variables, compared using the Kruskal–Wallis H test (^a^), and as n (%) for categorical variables, compared using the chi-square test (^b^). *p* < 0.05 was considered statistically significant.

BMI, body mass index; HC, healthy control; HPV, human papillomavirus infection; LSIL, low-grade squamous intraepithelial lesion; HSIL, high-grade squamous intraepithelial lesion; CC, cervical cancer.

### 3.2 Sequencing results

A total of 11,129,640 high-quality clean reads were obtained from 125 samples, with a mean of 89,037 ± 42,084 clean reads per sample. A total of 2,405 amplicon sequence variants (ASVs) were detected, among which 1,096 were shared by all five groups. The total ASV counts for each group were as follows: 1,651 in the HC group, 1,812 in the HPV group, 1,791 in the LSIL group, 1,844 in the HSIL group and 1,891 in the CC group. The mean number of observed ASVs was 283.04 for HC, 273.04 for HPV, 327.76 for LSIL, 326.28 for HSIL and 277.64 for CC. Overall, the HC group had the lowest cumulative number of ASVs, whereas the CC group had the highest (**Figure 1A**). Taxonomic annotation identified 13 phyla, 18 classes, 35 orders, 63 families, 106 genera and 289 species. The rarefaction curves of all groups plateaued and overlapped with one another, suggesting that the sequencing depth was adequate to cover the vast majority of microbial species in the samples (**Figure 1B**).

**Figure 1:**
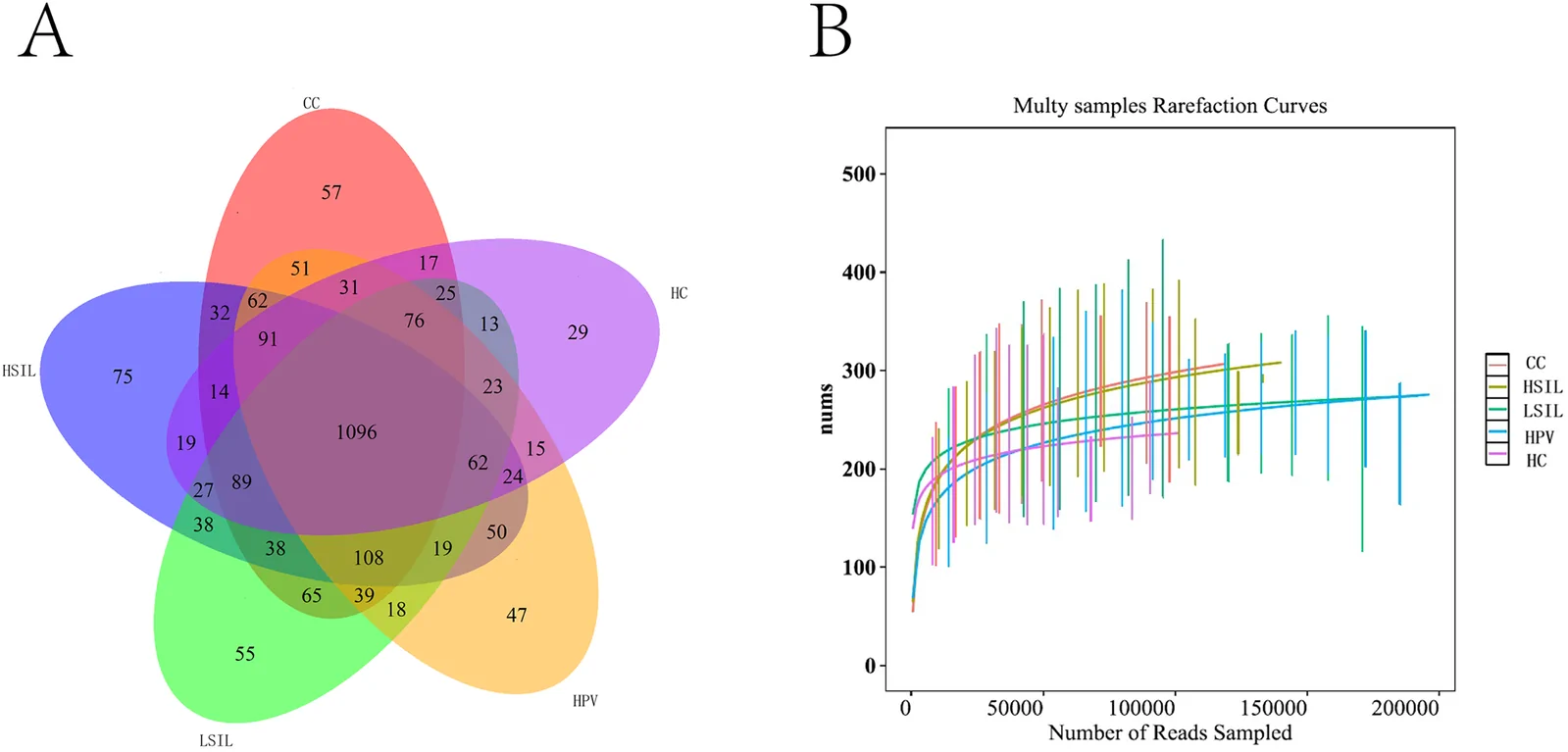
Sequencing depth and shared ASVs of the vaginal microbiota. **(A)** Flower plot depicting the numbers of unique and shared ASVs among the five groups (HC, HPV, LSIL, HSIL, and CC). Each petal represents a single group. **(B)** Rarefaction curves showing observed ASVs as a function of sequencing reads. All curves plateaued and overlapped across samples. ASV, amplicon sequence variant; HC, healthy control; HPV, human papillomavirus infection; LSIL, low-grade squamous intraepithelial lesion; HSIL, high-grade squamous intraepithelial lesion; CC, cervical cancer.

### 3.3 Characterization of vaginal microbial communities across different cervical lesion stages

Full-length 16S rRNA gene sequencing was performed on the Nanopore platform to characterize the taxonomic abundance of vaginal microbiota in participants across the five groups. Alpha diversity of vaginal microbiota was analyzed at the ASV level among the HC, HPV, LSIL, HSIL and CC groups. Statistically significant intergroup differences were observed for the Chao1, ACE indices and Good’s coverage (*p* = 0.0223, *p* = 0.0333, and *p* < 0.001, respectively). By contrast, no significant differences were detected for Richness, Shannon, Simpson, InvSimpson and Pielou indices (all *p* > 0.05; see **Supplementary Table S1**).

Principal coordinate analysis (PCoA) based on Bray-Curtis distance combined with permutational multivariate analysis of variance (PERMANOVA) was used to evaluate overall structural differences in vaginal microbiota across different stages of cervical lesions. At the ASV level, PERMANOVA indicated no significant differences in microbial composition among the five groups (*R*^2^ = 0.038, *p* = 0.142), and the PCoA plot showed substantial overlap of samples from all groups (**Figure 2A1**). Considering that the high-dimensional and sparse nature of ASV data may obscure overall shifts in microbial community structure, we further performed PERMANOVA at six taxonomic levels: phylum, class, order, family, genus and species (**Supplementary Table S2**). Highly significant intergroup differences in microbial composition were identified at the phylum level (*R*² = 0.105, *p* = 0.001), and partial separation between the CC and HC groups was observed in the corresponding PCoA plot (**Figure 2A2**). Significant differences were also present at the class, order, family and species levels (all *p* < 0.05), while no significant difference was found at the genus level (*p* > 0.05). The *R*^2^ values decreased progressively as taxonomic resolution increased. This finding suggests that microbiota changes linked to cervical lesions are primarily driven by fluctuations in the relative abundance of dominant phyla, including *Bacillota* and *Bacteroidota*. Heatmaps were generated based on species-level abundance profiles to visualize differences in microbial community structure across the five groups. *Lactobacillus iners* was the most abundant species overall, and the color gradient represented normalized species abundance (**Figure 2B**). Detailed abundance information of bacterial genera and species is shown in **Figures 2C1, 2C2**.

**Figure 2:**
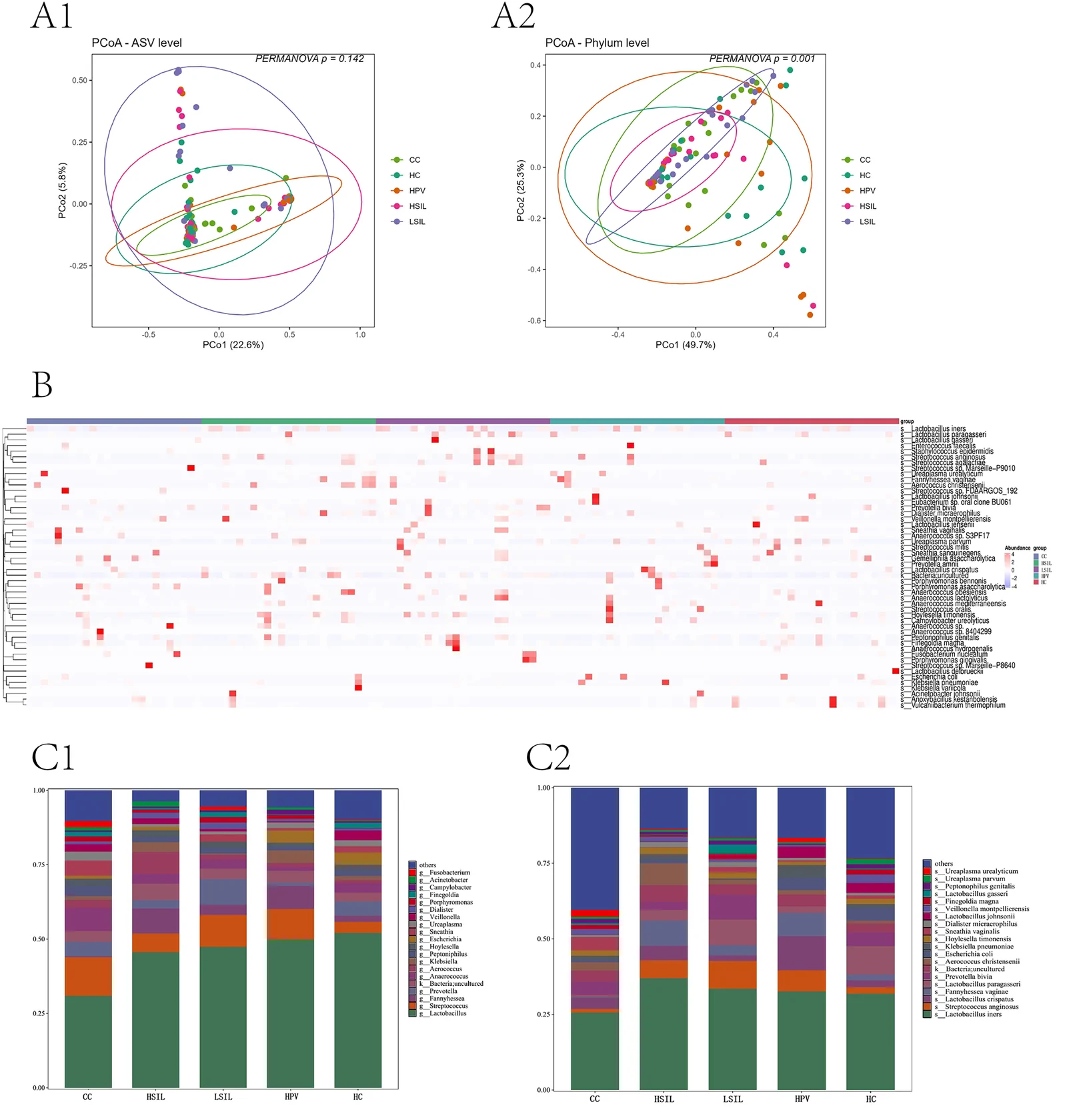
β-diversity and taxonomic composition of the vaginal microbiota. **(A1)** Principal-coordinate analysis (PCoA) plot based on amplicon sequence variant (ASV)-level microbial profiles. **(A2)** Principal-coordinate analysis (PCoA) plot based on phylum-level microbial profiles. **(B)** Heatmap of the top 50 microbial species with rows normalized by Z-score transformation. **(C1)** Stacked bar plot at the genus level across the five clinical groups. **(C2)** Stacked bar plot at the species level across the five clinical groups. ASV, amplicon sequence variant; PCoA, principal coordinate analysis. HC, healthy control; HPV, human papillomavirus infection; LSIL, low-grade squamous intraepithelial lesion; HSIL, high-grade squamous intraepithelial lesion; CC, cervical cancer.

Dirichlet multinomial mixture (DMM) unsupervised clustering was performed to partition all samples into three community types (Cluster 1–3) based on microbial composition and relative abundance (**Figure 3A**; **Supplementary Table S3**). Cluster 1 was characterized by *Fannyhessea vaginae*, *Streptococcus anginosus*, *Prevotella bivia*, *L. crispatus* and *L. iners*, showing a coexistence pattern of lactobacilli and facultative anaerobes. Cluster 2 was predominantly dominated by *L. iners*, with its average relative abundance reaching 90.9%. Cluster 3 was dominated by *L. paragasseri* and *L. gasseri*, whose combined relative abundance was over 55%. For community type distribution across groups, 80% of samples in the CC group were assigned to Cluster 1. Cluster 3 accounted for 20% of samples in both the HC and LSIL groups. The chi-square test revealed no statistically significant intergroup difference (*p* = 0.178). Nevertheless, an enrichment trend of Cluster 1 was observed in the CC group, suggesting that this community type may be associated with CC progression. Further validation using larger sample sizes is required. **Figure 3B** shows a Sankey diagram illustrating representative species belonging to the dominant phyla *Bacillota* and *Bacteroidota* across the five groups. *Bacillota* predominated in all groups, yet its dominant species varied by cohort: the HC group was dominated by *Streptococcus* sp. Marseille-P8640, the HPV group by *L. crispatus*, the LSIL group by *L. paragasseri*, the HSIL group by *L. iners*, and the CC group by *L. johnsonii*. For the phylum *Bacteroidota*, *P. bivia* prevailed in HC, *P. vaginalis* in HPV, and *P. gingivalis* in LSIL, HSIL and CC.

**Figure 3:**
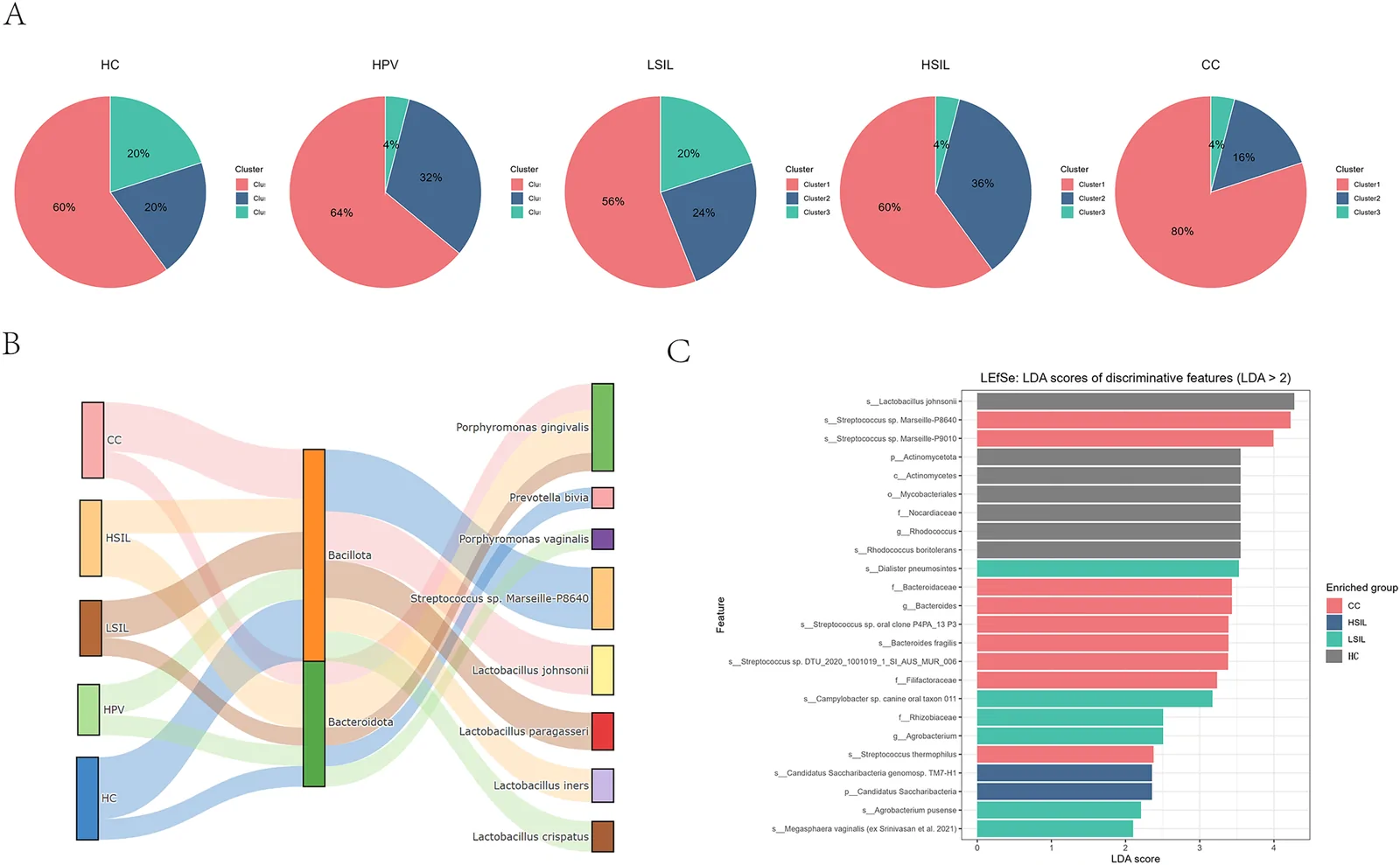
Community clustering, compositional flow, and biomarker taxa. **(A)** DMM-based clustering showing the proportion of three community state types (Cluster 1–3) across the five groups. **(B)** Sankey diagram illustrating the compositional flow from clinical groups to dominant phyla and representative species. **(C)** LEfSe bar plot displaying discriminatory taxa with LDA > 2 and *p* < 0.05. DMM, Dirichlet multinomial mixture; LEfSe, linear discriminant analysis effect size; LDA, linear discriminant analysis. HC, healthy control; HPV, human papillomavirus infection; LSIL, low-grade squamous intraepithelial lesion; HSIL, high-grade squamous intraepithelial lesion; CC, cervical cancer.

To screen microbial biomarkers characteristic of distinct cervical lesion stages, LEfSe analysis was conducted across all taxonomic ranks (phylum to species) with thresholds LDA > 2 and *P* < 0.05. We identified 28 differential taxonomic units, 24 of which received definitive taxonomic annotations (**Figure 3C**). *L. johnsonii* (LDA = 4.27) and taxa under *Actinomycetota* were enriched in HC; *Dialister pneumosintes*, *Campylobacter* sp. and other taxa in LSIL; the phylum and corresponding species of *Candidatus Saccharibacteria* in HSIL; and multiple *Streptococcus* spp., *Bacteroidaceae a*nd *B. fragilis* in CC. No differential biomarkers were detected in the HPV group. *Post-hoc* Dunn’s tests with Bonferroni correction confirmed significantly higher abundance of *L. johnsonii* in HC, *Ca. Saccharibacteria* in HSIL, *Campylobacter* sp. and *L. paragasseri* in LSIL, as well as *Streptococcus* spp. and *B. fragilis* in CC (all adjusted *P* < 0.05).

### 3.4 Community structure, differential species, and co-occurrence network of the vaginal microbiota across different stages of cervical lesions

To identify differential bacterial species across distinct cervical lesion stages, Mann-Whitney U tests were performed for four core pairwise comparisons (HC vs CC, HC vs HSIL, HPV vs CC, LSIL vs HSIL) using species-level abundance data, with FDR multiple testing correction (adjusted *q* < 0.05). We identified 35, 24, 24 and 13 differentially abundant species (DABs; **Figure 4A**) from the four comparisons respectively, which mainly belonged to the phyla *Bacillota*, *Actinomycetota* and *Bacteroidota*. The HC vs CC comparison yielded the largest number of DABs (35), 23 of which were classified as *Bacillota*. In the CC group, lactobacilli including *L. johnsonii* and *L. delbrueckii* were depleted, while opportunistic pathogens such as *D. pneumosintes*, *B. fragilis* and *Peptoniphilus* sp. were enriched. Twenty-four DABs were detected in the HC vs HSIL comparison, five of which fell under *Pseudomonadota*, implying that HSIL may be accompanied by the invasion of facultative anaerobes. The HPV vs CC comparison also produced 24 DABs, reflecting evident microbiota remodeling during progression from HPV infection to CC, characterized by reduced lactobacilli and elevated pathogens. Only 13 DABs were found in the LSIL vs HSIL comparison, nine of which were *Bacillota*, indicating milder microbial community shifts during progression from low- to high-grade lesions.

**Figure 4:**
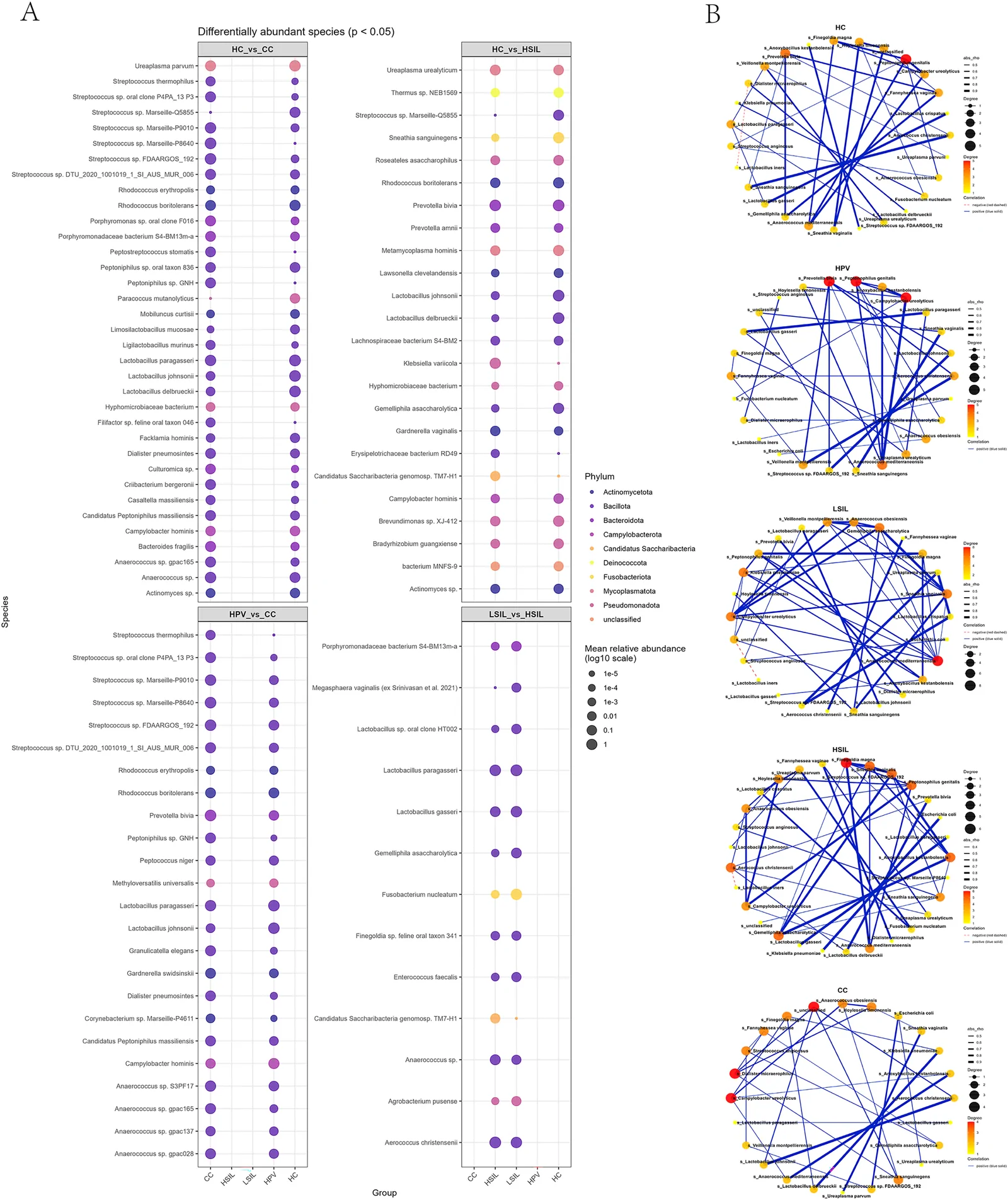
Differential species and correlation networks. **(A)** Bubble plot showing differentially abundant bacterial taxa identified by Mann-Whitney U tests (FDR < 0.05) in four pairwise comparisons. Bubble size indicates mean relative abundance, and bubble color represents phylum taxonomy. **(B)** Spearman correlation networks of the top 30 species constructed under the threshold of |ρ| > 0.2 and *p* < 0.05. Significant species pairs in HC, HPV, LSIL, HSIL, and CC groups were 30, 32, 41, 41, and 28, respectively. Solid edges indicate positive correlations, and dashed edges indicate negative correlations. FDR, false discovery rate. HC, healthy control; HPV, human papillomavirus infection; LSIL, low-grade squamous intraepithelial lesion; HSIL, high-grade squamous intraepithelial lesion; CC, cervical cancer.

Spearman correlation analysis was conducted on the top 30 shared abundant species across all five groups. Correlations satisfying |*r*| > 0.2 and FDR-adjusted *q* < 0.05 were considered significant. The counts of significant correlated pairs per group were as follows: HC = 30, HPV = 32, LSIL = 41, HSIL = 41, CC = 28. Co-occurrence network complexity rose at the LSIL and HSIL stages and declined in the CC group (**Figure 4B**). Stable positive correlations were detected among opportunistic pathogens. For instance, *Campylobacter ureolyticus* and *Anaerococcus mediterraneensis* displayed strong positive correlations in all five groups (*r* = 0.64–0.96). *Finegoldia magna* and *Peptoniphilus genitalis* consistently co-occurred in the HC, LSIL and HSIL groups (*r* > 0.78). Interspecies associations among lactobacilli shifted dynamically with lesion progression. In the HC group, *L. crispatus* was strongly positively correlated with *L. gasseri*, whereas a negative correlation existed between *L. iners* and *D. micraerophilus*. In the HPV group, the positive association between *L. johnsonii* and *Streptococcus* sp. was strengthened. A high correlation coefficient was observed between *L. crispatus* and *Ureaplasma parvum* in the LSIL group. In the HSIL group, *L. paragasseri* and *L. gasseri* maintained strong positive correlation, while *L. iners* correlated negatively with *Aerococcus christensenii*. In the CC group, an extremely strong positive correlation was found between *L. johnsonii* and *A. christensenii*, and *L. delbrueckii* was positively correlated with *Klebsiella pneumoniae*. Lesion-specific strong pathogen interactions were exclusively observed in HSIL and CC samples: *Escherichia coli* strongly correlated with *K. pneumoniae* in HSIL; unique robust correlations in CC included *K. pneumoniae* with *L. delbrueckii*, as well as *Sneathia vaginalis* with *U. parvum*.

### 3.5 Functional prediction and species-pathway correlation analysis

#### 3.5.1 Functional prediction

Functional profiles were predicted using PICRUSt2, revealing that Metabolism represented the dominant KEGG Level 1 functional category across all five groups, accounting for over 70% of total functions (**Figure 5A**). Although the overall composition of Level 1 categories was comparable among groups, Kruskal-Wallis tests detected significant intergroup differences for all categories (FDR-adjusted *P* < 0.001, **Supplementary Table S4**). At the KEGG Level 3 pathway level, 92 pathways exhibited significantly differential abundances across groups (FDR-adjusted *P* < 0.05). Twenty-two pathways were enriched in the HPV group, including ABC transporters and the Bacterial secretion system. The LSIL group contained the largest number of enriched pathways (60), covering alanine, aspartate and glutamate metabolism, glycolysis/gluconeogenesis and ribosome-related functions. Seven pathways were enriched in the HSIL group, predominantly butanoate metabolism and tryptophan metabolism. No pathways reached the highest mean abundance in either the HC or CC group. The heatmap of these 92 differential pathways (**Figure 5B**) illustrated distinct functional patterns: most pathways showed high abundance in the LSIL group, overall low abundance in the HC group, and prominent enrichment of secretion-associated pathways in the HPV group.

**Figure 5:**
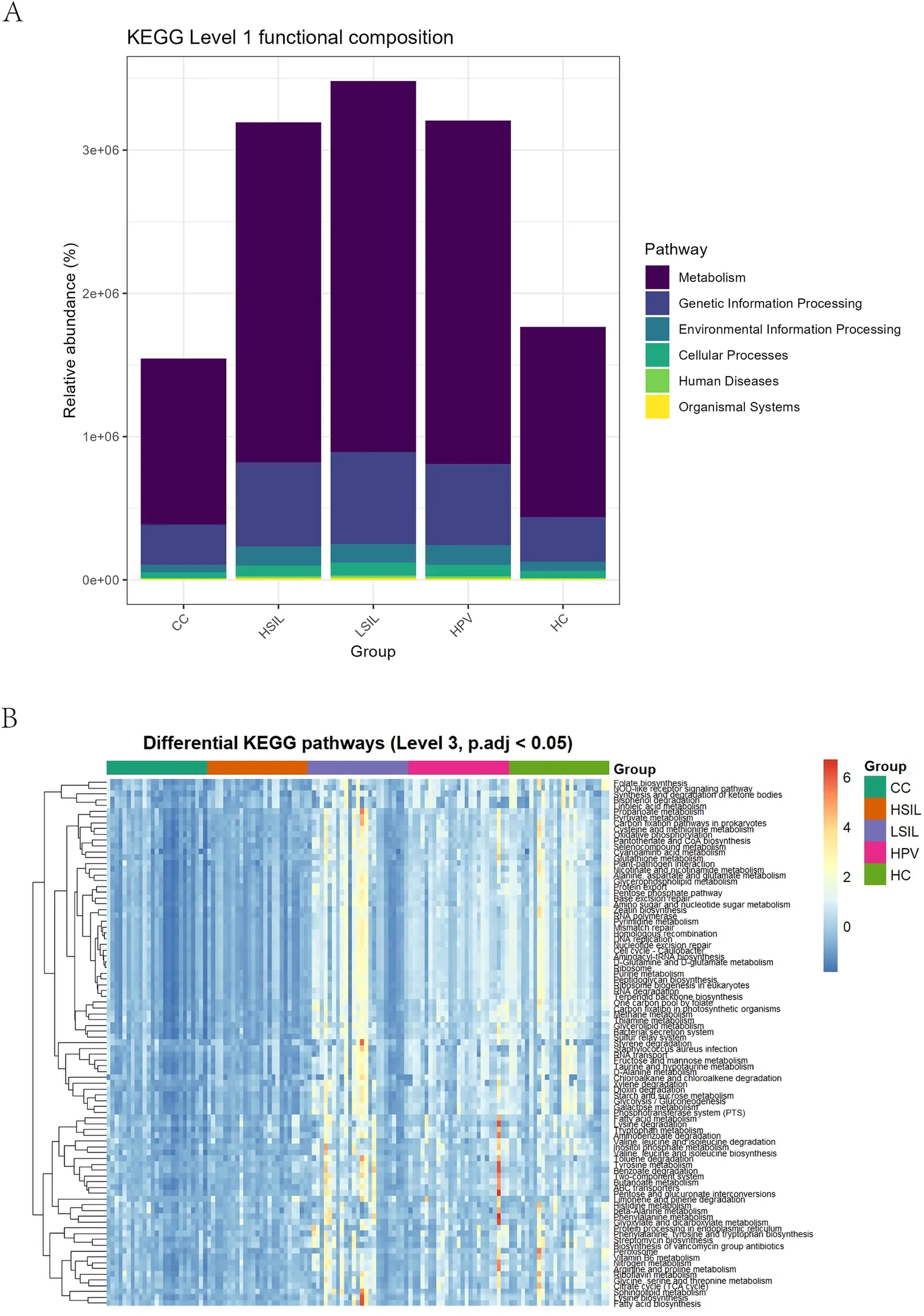
Functional prediction of the vaginal microbiota. **(A)** Stacked bar plot showing the relative abundance of microbial functional pathways at the KEGG Level 1. Statistical differences among groups were evaluated using the Kruskal–Wallis test (FDR < 0.001). **(B)** Heatmap illustrating the differential KEGG Level 3 pathways with adjusted *p* values (*p*.adj < 0.05, FDR-adjusted). Color intensity was normalized by row-wise Z-score. KEGG, Kyoto Encyclopedia of Genes and Genomes; FDR, false discovery rate. HC, healthy control; HPV, human papillomavirus infection; LSIL, low-grade squamous intraepithelial lesion; HSIL, high-grade squamous intraepithelial lesion; CC, cervical cancer.

#### 3.5.2 Species-pathway correlations

Spearman’s correlation analysis with FDR multiple testing correction was performed, and correlations satisfying |ρ| > 0.5 and FDR-adjusted *p.*adj < 0.05 were defined as statistically significant. A total of 17 significant species-pathway correlation pairs were identified (**Supplementary Table S5**). *Rhodococcus boritolerans*, which was abundant in the HC group, was strongly positively correlated with limonene and pinene degradation (|ρ| = 0.742). *L. iners* displayed bidirectional correlations with diverse pathways. Its positive associations included styrene degradation, linoleic acid metabolism and others (|ρ| ranging from 0.50 to 0.61), while negative correlations were observed with vancomycin group antibiotics biosynthesis and the citrate cycle (TCA cycle), among others (|ρ| ranging from −0.64 to −0.50). *Brevundimonas* sp. XJ-412 was significantly negatively correlated with D-alanine metabolism, zeatin biosynthesis and other pathways (|ρ| ranging from −0.52 to −0.51). *D. micraerophilus* showed a positive correlation with phenylalanine, tyrosine and tryptophan biosynthesis (|ρ| = 0.50).

## 4 Discussion

This study characterized changes in the vaginal microbiota (VMB) across the continuum from high-risk HPV infection to cervical cancer among postmenopausal women, with the aim of informing clinical management for this patient population. This is the first study in Xinjiang, China to profile vaginal microbiota associated with high-risk HPV infection in postmenopausal women using Nanopore full-length 16S rRNA sequencing. Previous research has demonstrated that age over 60 years is a risk factor for HSIL and cervical cancer (Han et al., 2026). Multiple studies have documented a high prevalence of persistent HPV infection in postmenopausal women. Compared with other epidemiological factors, menopausal status exerts a stronger influence on vaginal microecological balance in HPV-positive individuals (Smith et al., 2004; So et al., 2024). It has also been reported that menopausal status acts as an independent predictor of persistent HPV infection following surgery for high-grade cervical lesions (García-Piñeres et al., 2006). Building on these findings, we recruited postmenopausal women as participants and systematically explored the associations between vaginal microbiota and high-risk HPV infection, cervical lesions of varying grades, as well as cervical cancer.

Species-level analysis indicated that *L. iners* was the predominant species across most samples. Microbial community composition changed substantially as cervical lesions advanced. In the CC group, the dominance of *L. iners* decreased significantly, while the abundances of opportunistic pathogens such as *P. bivia* and *S. vaginalis* increased. The HSIL and LSIL groups were still predominated by *L. iners*, yet enriched with the facultative anaerobe *S. anginosus* and obligate anaerobe *F. magna*. The HC group exhibited a relatively stable microbiota structure, with *L. iners* as the absolute dominant species and other taxa present at low proportions. These results suggest progressive vaginal dysbiosis throughout cervical lesion development. The elevated abundance of opportunistic pathogens and declined dominance of lactobacilli may collectively drive disease progression. Our observations are in line with previous reports (Kyrgiou and Moscicki, 2022; Huang et al., 2024; Pu et al., 2025). The loss of lactobacilli and overgrowth of opportunistic pathogens likely disrupt vaginal microecological homeostasis and further facilitate the progression of cervical lesions. Alpha diversity analysis was performed to evaluate vaginal microbiota alterations in postmenopausal women during the progression from high-risk HPV infection to cervical cancer. No significant intergroup differences were found in the Shannon and Simpson indices, indicating that the overall diversity and species evenness of vaginal microbiota remained stable across different stages of cervical lesions. Previous research has demonstrated that postmenopausal women harbor a lower total bacterial load and more evenly distributed vaginal microbiota than premenopausal women (Gliniewicz et al., 2019), which may partially account for the stable patterns of the Shannon and Simpson indices in the present study. Significant differences were detected in the Chao1 and ACE indices, which reflect species richness, as well as Good’s coverage among the five groups. Relative to the healthy control group, the total number of microbial species gradually increased from HPV infection to LSIL and HSIL stages. Although species richness declined at the cervical cancer stage, it was still higher than that in the HPV group. This trend is in line with prior findings (Nieves-Ramírez et al., 2021; Wang et al., 2022; Pu et al., 2025; Yang et al., 2025). It suggests that high-risk HPV infection and cervical lesions may induce dramatic fluctuations or even depletion of certain bacterial taxa, consequently altering community richness.

*P. bivia* is an anaerobic species linked to bacterial vaginosis. It produces lipopolysaccharides and is capable of adhering to and invading human cervical epithelial cells. Accumulating evidence has associated this bacterium with persistent HPV infection (Bradshaw and Sobel, 2016; Chao et al., 2020). Increased relative abundance of *Sneathia sanguinegens* in the vaginal microbiome has been correlated with the presence of clue cells and bacterial biofilms, indicating that the genus *Sneathia* plays a vital role in vaginal biofilm formation (Gottschick et al., 2017). Multiple studies have reported higher abundance of *Sneathia* spp. in HPV-positive women and patients with CIN of varying grades (Lee et al., 2013; Mitra et al., 2015; Audirac-Chalifour et al., 2016; Łaniewski et al., 2018; Chen et al., 2019; Onywera et al., 2019; Zhou et al., 2019). Furthermore, *S. vaginalis* can adhere to malignant cervical epithelial cells (Harwich et al., 2012). Its secreted toxic products may directly alter host tissue properties and disrupt the cervical microenvironment. A systematic review (Wu et al., 2022) demonstrated that streptococci were detected at significantly higher rates in the vaginal microbiota of patients with HPV infection, LSIL, HSIL and CC. Consistent with this, our LEfSe analysis identified significant enrichment of *Streptococcus* sp. Marseille-P8640 and *Streptococcus* sp. Marseille-P9010 in CC samples. Infections caused by streptococci and other bacteria can aggravate chronic inflammation, which in turn creates a permissive niche for HPV persistence and malignant progression (Yang et al., 2025). Streptococci modulate local inflammatory responses and may influence the persistence and progression of HPV infection, though the underlying mechanisms remain to be elucidated.

The role of *L. iners* within the vaginal microbiota appears multifaceted. It can dominate the vaginal niche in healthy women but is also frequently detected under conditions of microbial dysbiosis such as bacterial vaginosis, even following antimicrobial treatment (Ferris et al., 2007; Goodfellow et al., 2021; Zheng et al., 2021; Petrova et al., 2017; Huang et al., 2024). *L. iners* carries one of the smallest genomes among vaginal lactobacilli (~1.3 Mbp). Notably, it lacks the gene encoding D-lactate dehydrogenase and therefore synthesizes only L-lactic acid, without producing D-lactic acid. Since D-lactic acid exhibits stronger antimicrobial activity against pathogenic bacteria than L-lactic acid (Tachedjian et al., 2017), this metabolic limitation may impair the capacity of *L. iners*-dominated communities to maintain an acidic vaginal environment, conferring weaker host-protective effects relative to *L. crispatus* (Srinivasan et al., 2012; Zheng et al., 2021). In addition, the *L. iners* genome encodes inerolysin, a pore-forming toxin homologous to the hemolysin produced by *Gardnerella vaginalis*, which may damage host epithelial cells under certain microenvironmental conditions (Petrova et al., 2017).

Previous work has demonstrated that communities dominated by *L. iners* are more prone to transition toward a BV-like state compared with *L. crispatus*-dominated microbiota (Witkin et al., 2013; Mortaki et al., 2020; Xu et al., 2022). Even after clinical treatment for bacterial vaginosis, the vaginal ecosystem often fails to shift from an *L. iners*-predominant profile back to an *L. crispatus*-dominated state. Furthermore, *L. iners* is closely linked to HPV infection, yet published findings remain inconsistent. While several studies have implied a potential role of *L. iners* in facilitating high-risk HPV clearance (Usyk et al., 2020), a longitudinal cohort by Shi et al. (2022) reported a negative correlation between *L. iners* abundance and high-risk HPV clearance among conservatively-managed patients with high-risk HPV related cervical lesions. Such discrepancies across studies may originate from differences in participants’ hormonal status (reproductive-age versus postmenopausal women), enrolment criteria, and sample-collection procedures.

This study, based on a cohort of postmenopausal women, found that *L. iners* was a dominant bacterial species across all five groups. Its abundance fluctuated markedly along the progression of cervical lesions: it remained high in the HC, HPV, LSIL and HSIL groups, but decreased sharply in the CC group, suggesting that the local microenvironment of cervical cancer may be hostile to *L. iners* colonization. Furthermore, a negative correlation between *L. iners* and anaerobic bacteria was detected only in lesion-affected groups (LSIL/HSIL/CC), implying potential antagonism between *L. iners* and opportunistic pathogens during lesion progression.

Accumulating in-vitro and clinical findings have advanced our understanding of the potential role of *L. iners* in cervical neoplasia. L-lactate produced by intratumoral *L. iners* can drive tumor metabolic reprogramming and induce chemoradiotherapy resistance in cervical cancer cells (Johnston and Bullman, 2024). Beyond acting as an energy substrate for tumor cells, L-lactate can suppress T-cell function, foster regulatory T-cell differentiation, and dampen host anti-tumor immune responses. These observations indicate that *L. iners* is not merely a passive bystander but may actively contribute to disease progression and therapeutic resistance through metabolic modulation (Liu and Cao, 2024).

Spearman correlation analysis in the present study demonstrated that *L. iners* abundance was significantly correlated with multiple KEGG functional pathways. *L. iners* may modulate the cervicovaginal microenvironment in postmenopausal women with cervical lesions by repressing energy-metabolism-related pathways and up-regulating inflammatory signaling cascades. KEGG functional annotation showed that metabolic pathways represented the most predominant functional category in all five groups, and the LSIL stage may serve as a critical turning point for functional remodeling of the vaginal microbiota. Collectively, *L. iners* displays pleiotropic effects during cervical lesion progression. Under healthy conditions, it acts as a normal vaginal commensal; nevertheless, during lesion development, its metabolic by-products, particularly L-lactate, may facilitate disease progression by reshaping the local microenvironment.

Several limitations of the present study need to be addressed. First, as a cross-sectional investigation, this study cannot clarify the causal relationship between vaginal microbiota dynamics and the progression of cervical lesions. Further longitudinal cohort studies with long-term monitoring of microbial profiles in HPV-infected populations are necessary to obtain more solid evidence for causality. Second, we detected numerous significant correlations among bacterial species, yet the underlying interaction mechanisms remain unclear due to insufficient relevant fundamental research. Third, although the associations of *L. iners*, *P. bivia* and other taxa with HPV infection and cervical lesions were confirmed, their detailed functional mechanisms have not been fully explored. Follow-up experiments are required to elucidate the molecular regulatory mechanisms by which these microorganisms and their metabolites contribute to persistent HPV infection, as well as the onset and progression of LSIL and cervical cancer.

## 5 Conclusions

In this study, full-length 16S rRNA sequencing based on the Nanopore platform was performed to characterize dynamic changes of vaginal microbiota during the progression from HPV infection to cervical cancer. No significant differences in overall microbial structure were observed at the ASV level, whereas remarkable remodeling occurred at the phylum level and in functional pathways. With the aggravation of cervical lesions, opportunistic pathogens including *D. pneumosintes* and *B. fragilis* were enriched, while certain lactobacilli such as *L. johnsonii* and *L. delbrueckii* were depleted. The complexity of microbial co-occurrence networks peaked at the LSIL and HSIL stages and decreased in cervical cancer. Functionally, the LSIL group harbored the largest number of enriched metabolic pathways. *L. iners* exhibited dual correlations with diverse pathways, indicating its potential role as a functional regulator in disease progression. These findings provide valuable clues for identifying microbial biomarkers and exploring the underlying mechanisms of cervical lesions.

## Data Availability

The raw sequence data reported in this paper have been deposited in the Genome Sequence Archive (GSA) in National Genomics Data Center, China National Center for Bioinformation / Beijing Institute of Genomics, Chinese Academy of Sciences (GSA: CRA050773; https://ngdc.cncb.ac.cn/gsa).
